# University physical education course experience and resilience-based psychological recovery capacity among students in Chinese higher education: indirect associations through emotion regulation, exercise self-efficacy, and peer connectedness

**DOI:** 10.3389/fpsyg.2026.1908934

**Published:** 2026-10-15

**Authors:** Wendong Yang, Liangfang Meng

**Affiliations:** 1 School of Physical Education, Hubei University of Arts and Science, Xiangyang, Hubei, China; 2 School of Health Management, Xianning Vocational Technical College, Xianning, Hubei, China

**Keywords:** embodied learning, emotion regulation, exercise self-efficacy, peer connectedness, psychological recovery capacity, structural equation modeling, student mental health, university physical education

## Abstract

University students face increasing psychological pressure, yet formal mental health services often remain limited in reach and depend on active help-seeking. This study examines university physical education (PE) as an educational context whose perceived qualities may be associated with psychological resources relevant to adaptation under stress. Using cross-sectional survey data from 831 Chinese college students, we examined whether perceived PE course experience was associated with resilience-based psychological recovery capacity and whether this association was indirectly linked through emotion regulation, exercise self-efficacy, and peer connectedness. The retained measurement specification represented PE course experience as a second-order construct comprising teacher support, mastery climate, embodied engagement, emotional release, and peer interaction. Structural equation modeling indicated that PE course experience was positively associated with resilience-based psychological recovery capacity and with all three proposed indirect-linking variables. Bootstrapping results supported significant indirect associations through emotion regulation, exercise self-efficacy, and peer connectedness, with exercise self-efficacy showing the largest estimated indirect association. The association remained significant after controlling for perceived stress and extracurricular exercise frequency. These findings link perceived PE course qualities with resilience-based adaptive capacity, conditional on the measured covariates. They do not isolate curriculum effects from physical-activity exposure. Because the design is cross-sectional, the findings should be interpreted as theoretically grounded associations rather than causal effects.

## 1 Introduction

Universities are facing a widening gap between rising student psychological distress and the limited reach of formal mental health services. Across different higher education systems, university students report increasing levels of stress, anxiety, depressive symptoms, loneliness, and uncertainty about the future. Evidence from the World Health Organization World Mental Health Surveys shows that mental disorders are highly prevalent among college students across countries, suggesting that student mental health is not a local or marginal issue but a global challenge for higher education (Auerbach et al., 2016). Longitudinal evidence from the United States further indicates increasing trends in mood and anxiety symptoms among undergraduates over the past decade (Duffy et al., 2019). Although universities have expanded counseling services and mental health education, the demand for psychological support continues to outpace institutional capacity (Lipson et al., 2019).

A central difficulty is that formal mental health services are often selective rather than universal. Counseling and clinical interventions can be effective, but they are typically resource-intensive and depend on students’ willingness to identify themselves as needing help. This creates a structural problem: students experiencing psychological distress may be precisely those least likely to seek formal support because of stigma, avoidance, limited awareness, or concerns about being judged (Eisenberg et al., 2009; Eisenberg et al., 2012). Higher education institutions therefore have reason to investigate additional support resources embedded in ordinary campus life, while their scalability, cost, reach, and perceived stigma require direct empirical evaluation. The present study examines whether students’ perceptions of an ordinary educational setting are associated with psychological resources relevant to adaptation under stress.

Against this background, university physical education (PE) courses represent an overlooked institutional resource. Unlike counseling services, PE courses do not require students to self-identify as psychologically vulnerable. Where PE courses are compulsory or credit-bearing, they offer a routinely organized setting in which to study student experiences. Comparative service reach was not evaluated in this study. More importantly, PE courses are not merely opportunities for physical activity. They are structured educational spaces involving bodily engagement, teacher guidance, peer interaction, emotional release, skill mastery, and repeated participation. PE courses provide an ordinary educational context that does not require formal mental-health help-seeking; whether students perceive this context as less stigmatizing was not examined in the present study.

Existing research provides strong evidence that physical activity and exercise are beneficial for mental health. Meta-analytic and review evidence has shown that exercise can reduce depressive symptoms and psychological distress, and that physical activity is associated with lower risk of depression and anxiety-related outcomes (Rebar et al., 2015; Pearce et al., 2022; Singh et al., 2023; Noetel et al., 2024). However, this literature has primarily conceptualized physical activity as an individual health behavior. Much less is known about university PE as an institutionalized educational setting. This distinction matters because universities cannot easily control students’ voluntary exercise habits, but they can design, organize, and improve PE courses. Therefore, the research gap is not whether exercise is generally good for mental health, but whether perceived university PE course experiences are associated with resilience-based psychological recovery capacity in high-pressure higher education contexts.

A smaller but growing course-level literature shows why the educational context itself warrants analysis. Among college PE students, perceived teacher autonomy support has been associated with psychological need satisfaction, autonomous motivation, wellness, learning, performance, and intentions to remain physically active (Behzadnia et al., 2018). Recent evidence from Chinese universities likewise links autonomy-supportive PE with motivation for physical activity and out-of-class activity (Guo et al., 2026), and links PE lesson activity with mental health and health-related behaviors (Xu et al., 2025). At the same time, ability exposure and evaluation anxiety in university PE have been associated with poorer psychological wellbeing, whereas PE belonging has shown favorable associations (Wang, 2026). Taken together, this literature indicates that PE is not psychologically uniform: its relevance depends on how teaching, evaluation, bodily participation, and social belonging are organized and perceived. The present study extends this course-level literature from motivation, activity, and wellbeing toward resilience-based psychological recovery capacity.

A second limitation concerns the outcomes typically emphasized in PE and mental health research. Studies in sport and exercise contexts often focus on physical fitness, motor competence, exercise participation, or clinical symptoms such as anxiety and depression. These outcomes are important, but they do not fully capture how students regain psychological resources under chronic academic and social pressure. Psychological recovery refers to the process through which individuals restore emotional balance, replenish psychological resources, and maintain adaptive functioning after stress. Recovery research has emphasized experiences such as relaxation, mastery, control, and psychological detachment as important pathways for restoring depleted resources (Sonnentag and Fritz, 2007). From the perspective of conservation of resources theory, stress becomes harmful when individuals lose or fail to regain valued psychological resources (Hobfoll, 1989). In this sense, university PE may be relevant not only because it reduces symptoms, but because its perceived qualities may be associated with adaptive capacity.

Three theoretically proposed indirect pathways motivate this study. Perceived opportunities for affective adjustment in PE are hypothesized to be associated with cognitive reappraisal, an emotion-regulation resource relevant to adaptation (Gross, 1998). Mastery-oriented PE experiences are hypothesized to be associated with exercise self-efficacy, consistent with self-efficacy theory and the competence component of self-determination theory (Bandura, 1997; Ryan and Deci, 2000). Supportive interactions in PE are hypothesized to be associated with peer connectedness, consistent with social support theory (Cohen and Wills, 1985). The present survey assesses these associations; it does not observe the proposed resource-development processes over time.

China provides a meaningful empirical context for examining this question. Chinese university students face intense academic competition, employment uncertainty, and rapid social change, all of which may heighten psychological pressure. Meta-analytic evidence indicates that depression among Chinese university students is a significant public health concern (Gao et al., 2020). However, the contribution of the present study is not limited to the Chinese context. Rather than treating China as an exceptional case, this study uses Chinese universities as an empirical setting for addressing a broader international question: is perceived university PE course experience associated with psychological resources relevant to adaptation under sustained stress?

This study makes three contributions. First, it shifts the focus from physical activity as an individual behavior to students’ perceived experience of university PE as an organized educational setting. Second, it advances resilience-based psychological recovery capacity as a meaningful outcome in PE research, moving beyond traditional emphases on physical fitness and clinical symptoms. Third, it develops and tests an indirect-association model in which perceived PE course experience is associated with psychological recovery through emotion regulation, exercise self-efficacy, and peer connectedness. The intended contribution is therefore not to claim that PE can replace counseling or clinical care, or that institutional design has been causally verified, but to examine associations consistent with a potentially recovery-supportive educational context. Preventive effects, stigma reduction, and scalability require prospective implementation research.

## 2 Conceptual framework and hypotheses

### 2.1 University PE course experience as a multidimensional educational context

This study conceptualizes university physical education course experience as students’ perceived quality of engagement in PE courses, including structured bodily participation, supportive teaching, mastery-oriented learning, emotional release, and peer interaction. This definition differs from general physical activity participation. Whereas physical activity is often treated as an individual health behavior, PE courses are institutionally organized educational settings in which students encounter repeated, structured, and socially embedded bodily experiences.

Physical-activity research provides evidence relevant to mental health (Rebar et al., 2015; Pearce et al., 2022; Singh et al., 2023; Noetel et al., 2024). The present framework focuses on an additional question: whether students’ perceptions of the teaching and social context of PE are associated with reported psychological resources. Supportive feedback, opportunities for mastery, and peer participation motivate this proposed association, but their effects on resource development are not observed in the present design.

The proposed construct differs in referent and scope from several neighboring constructs, although substantial overlap remains possible. Perceived autonomy support concerns support for choice and volition from particular social agents (Hagger et al., 2007). Mastery climate emphasizes effort, learning, and self-referenced improvement (Ames, 1992); it is deliberately included as one PE experience dimension, not treated as wholly distinct from that dimension. Enjoyment concerns the positive affect associated with participation and has been examined separately from goals and motivation in school PE (Wang and Liu, 2007). Emotional Release, in contrast, concerns perceived relief from tension, although its items also contain affective self-reports and may overlap with enjoyment. Physical self-perceptions concern judgments of one’s body, competence, condition, and physical self-worth (Fox and Corbin, 1989), whereas exercise motivation concerns reasons for participation (Ryan and Deci, 2000). PE course experience is intended to integrate several course-referent perceptions, including students’ perceived emotional consequences of participation. This proposed scope is a conceptual rationale, not evidence of external discriminant validity or a distinction between statistical levels of analysis.

Research with Chinese college students has modeled perceived autonomy support in PE and autonomous motivation as separate constructs (Abula et al., 2020). This provides a relevant precedent for distinguishing perceptions of instruction from motivational regulation, but it does not validate the present PE course-experience measure or establish its incremental value over existing instruments.

### 2.2 Emotion regulation as a theoretically proposed pathway

Emotion regulation refers to the processes through which individuals influence the emotions they experience, when they experience them, and how these emotions are expressed or managed (Gross, 1998). In high-pressure higher education contexts, students are frequently exposed to academic demands, performance evaluation, interpersonal comparison, and uncertainty about future employment. Under these conditions, the ability to regulate emotional responses becomes central to psychological adaptation.

PE course experience is theorized to be associated with emotion regulation within embodied and affective course contexts. Opportunities for physical participation, changes in attentional focus, and perceived emotional release provide a rationale for examining cognitive reappraisal alongside course experience. The present measures capture students’ perceptions and reported regulatory tendencies; they do not observe physiological activation, immediate affective change, or the temporal development of regulation skills.

### 2.3 Exercise self-efficacy as a mastery-based pathway

Exercise self-efficacy refers to students’ belief in their ability to participate in, persist in, and benefit from physical activity. According to self-efficacy theory, mastery experiences are a primary source of perceived capability (Bandura, 1997). PE courses can provide such mastery experiences when students learn skills, complete physical tasks, overcome challenges, and perceive improvement in their own performance.

This pathway is also consistent with self-determination theory, which emphasizes competence, autonomy, and relatedness as basic psychological needs supporting motivation and wellbeing (Ryan and Deci, 2000). Supportive and mastery-oriented PE experiences are theoretically expected to be associated with stronger perceived competence and control. Such perceptions are relevant to broader adaptive capacity, although the present design does not establish their development or transfer beyond the PE setting over time.

### 2.4 Peer connectedness as a social recovery pathway

Peer connectedness refers to students’ perceived sense of belonging, acceptance, and supportive interaction with peers. Social support theory suggests that interpersonal support can buffer the negative effects of stress and protect psychological wellbeing (Cohen and Wills, 1985). For university students, peer relationships are especially important because much of their stress is embedded in shared academic, social, and developmental contexts.

PE courses may provide a distinctive environment for peer connectedness. Many PE activities involve cooperation, group practice, informal communication, and shared bodily experience. These interactions may create opportunities for students to feel recognized, included, and supported. Such support occurs through ordinary course participation rather than formal help-seeking; whether students perceive this context as less stigmatizing requires direct empirical evaluation.

### 2.5 Resilience-based psychological recovery capacity

The distinction between recovery and resilience requires explicit boundaries. Recovery is often conceptualized as a dynamic process through which depleted psychological resources are restored, whereas resilience is a comparatively durable capacity for adaptation under stress. The outcome in this study was measured with the CD-RISC-10 and is therefore defined as resilience-based psychological recovery capacity: a perceived adaptive capacity under sustained pressure, rather than momentary recovery after a single PE lesson. This term does not make resilience and recovery interchangeable; it identifies resilience as the measured macro-level indicator used in the present model.

This operational choice is especially relevant in high-pressure higher education contexts, where stress is chronic rather than episodic. Repeated positive PE experiences may be associated with more durable perceived adaptive capacity, but the present cross-sectional design cannot establish accumulation over time. For this reason, resilience is used as a macro-level indicator of resilience-based psychological recovery capacity rather than as a direct measure of immediate recovery state. This distinction narrows the theoretical claim of the study and avoids equating short-term recovery episodes with stable resilience.

### 2.6 Proposed model

Based on the above reasoning, this study proposes a parallel indirect-association model in which university PE course experience is associated with resilience-based psychological recovery capacity directly and indirectly through emotion regulation, exercise self-efficacy, and peer connectedness. Perceived stress and extracurricular exercise frequency are included as the primary covariates. Gender, grade level, and institution are described as sample characteristics rather than treated as core structural covariates (Figure 1).

**Figure 1:**
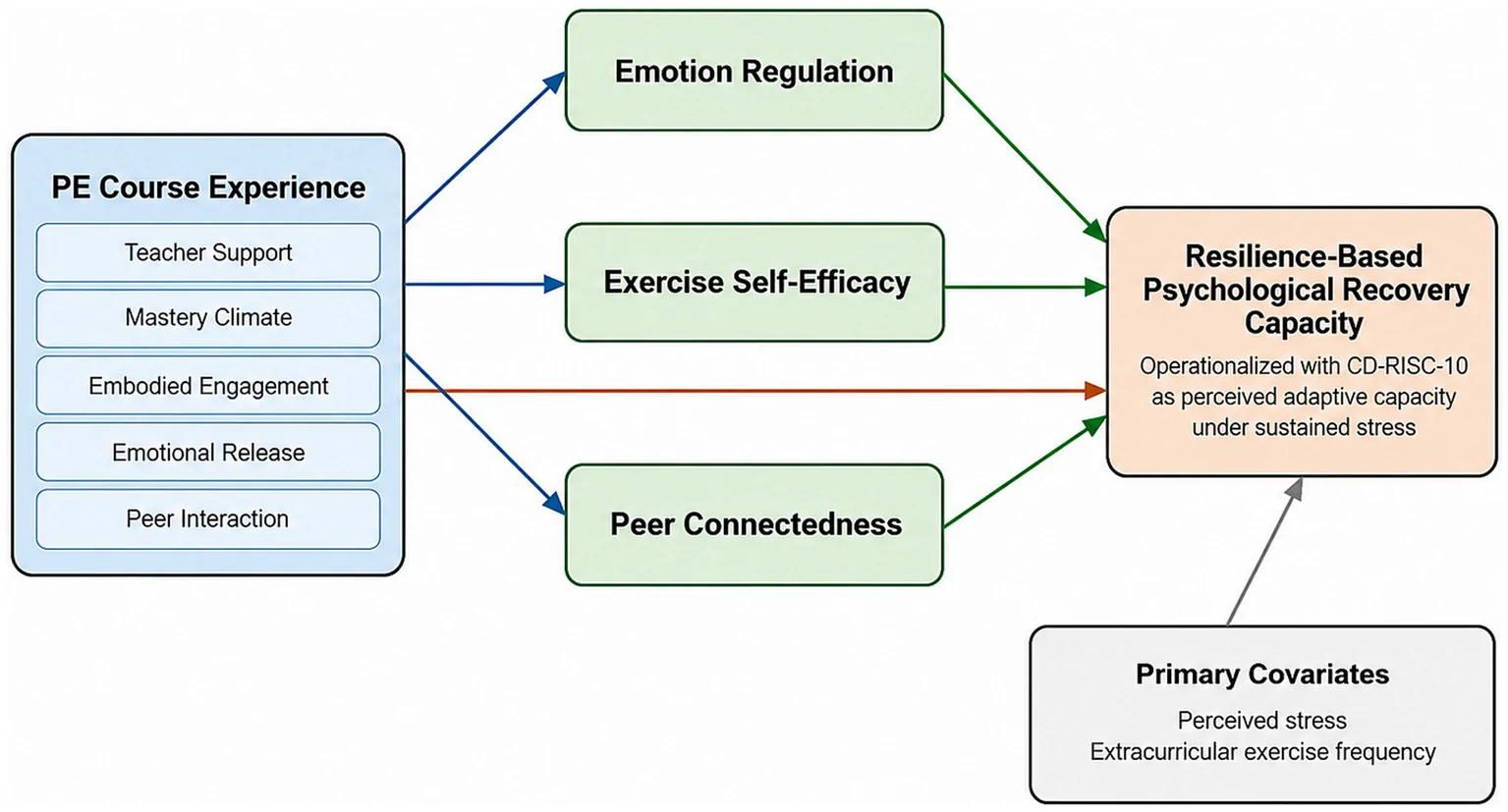
Proposed theoretical model linking perceived PE course experience to resilience-based psychological recovery capacity.

Accordingly, the following hypotheses are proposed:

*H1*. University PE course experience is positively associated with resilience-based psychological recovery capacity among university students.

*H2*. University PE course experience is positively associated with emotion regulation.

*H3*. University PE course experience is positively associated with exercise self-efficacy.

*H4*. University PE course experience is positively associated with peer connectedness.

*H5*. Emotion regulation forms a statistically significant indirect association between university PE course experience and resilience-based psychological recovery capacity.

*H6*. Exercise self-efficacy forms a statistically significant indirect association between university PE course experience and resilience-based psychological recovery capacity.

*H7*. Peer connectedness forms a statistically significant indirect association between university PE course experience and resilience-based psychological recovery capacity.

## 3 Materials and methods

### 3.1 Study design and participants

This study adopted a cross-sectional survey design to examine associations between perceived PE course experience and resilience-based psychological recovery capacity among Chinese higher-education students. Participants were full-time students in undergraduate or vocational-college programs who had completed at least one semester of PE courses. The final sample comprised 455 undergraduate students and 376 vocational-college students (*N* = 831).

Convenience and purposive sampling was used. PE teachers recruited students in intact PE classes and distributed a questionnaire QR code. Four institutions were included to provide institutional heterogeneity across undergraduate and vocational-college settings; this was a coverage decision rather than a probability-sampling design or a claim of population representativeness. Data were collected from March to May 2025 in Xiangyang and Xianning, Hubei Province. The final analytic sample included 831 respondents after eligibility, attention-check, and data-quality screening. Participation was voluntary and anonymous.

The study was reviewed and approved by the School of Physical Education, Hubei University of Arts and Science, on December 10, 2024. A formal ethics approval number was not issued by the approving unit. Participation was voluntary and anonymous, and all participants provided informed consent before completing the questionnaire.

The final sample size of 831 exceeded common recommendations for structural equation modeling with multiple latent variables and parallel indirect paths, providing adequate statistical power for confirmatory factor analysis, structural path testing, and bootstrapped indirect-effect estimation.

Critically, the temporal ordering in the model is theoretical rather than empirical. PE course experience was specified as the antecedent because it represents students’ evaluation of an institutional course context, whereas emotion regulation, exercise self-efficacy, peer connectedness, and resilience-based psychological recovery capacity represent psychological or social resources hypothesized to be supported by that context. This ordering is consistent with the conceptual model, but it should not be interpreted as longitudinal evidence.

### 3.2 Measures

All 53 focal scale items used a harmonized 5-point Likert-type agreement format from 1 = strongly disagree to 5 = strongly agree; 14 demographic, screening, and attention-check items brought the total questionnaire to 67 items. Construct scores were computed as item means after applicable reverse scoring. Because the harmonized format differs from the response metrics of some source instruments, it is treated as a study-specific measurement adaptation rather than assumed to be psychometrically neutral. Chinese-language forms based on established source scales were used where available, and the PE course experience items were developed for the university PE context. Scale provenance, scoring, and adaptation details are summarized in Table 1 and Supplementary Appendix C (Supplementary Table C1).

**Table 1:** Measurement sources and adaptation rationale.

Construct	Source or basis	Adaptation rationale
PE course experience	Context-specific scale developed from teacher support/autonomy support, mastery climate, recovery experience, and social support perspectives (Ames, 1992; Hagger et al., 2007; Sonnentag and Fritz, 2007; Cohen and Wills, 1985).	Items were written to capture PE as a course ecology: teacher support, mastery climate, embodied engagement, emotional release, and peer interaction.
Psychological recovery capacity	CD-RISC-10; Wang et al. (2010); Campbell-Sills and Stein (2007).	Used as a macro-level indicator of resilience-based recovery capacity under sustained stress, not as a momentary recovery-state measure.
Emotion regulation	ERQ cognitive reappraisal dimension; Wang et al. (2007); Gross and John (2003).	Reappraisal was retained because it best matches adaptive reinterpretation and emotional adjustment after stress.
Exercise self-efficacy	Self-efficacy theory and exercise self-efficacy measures; Bandura (1997); Marcus et al. (1992).	Items were adapted to university PE tasks and students’ confidence in using exercise participation to manage stress.
Peer connectedness	Social connectedness and social support perspectives; Cohen and Wills (1985); Lee and Robbins (1995).	Items were contextualized to classmates and interaction quality inside PE courses rather than general friendship networks.
Perceived stress	PSS-10; Lu et al. (2017); Cohen and Williamson (1988) for the original construct	Chinese-language adaptation; 1–5 agreement format; Items 4, 5, 7, and 8 reverse-scored

The PE course experience scale was context-specific and therefore requires further validation beyond the present sample. Established scales were used where available, and adaptations were made to align item wording with university PE course contexts.

For the context-specific PE course experience items and the contextualized exercise self-efficacy and peer connectedness items, item wording was developed in Chinese to match ordinary university PE course settings and then translated into English for reporting. Before the formal survey, six experts with backgrounds in physical education pedagogy and psychometrics evaluated the PE course experience items using a 4-point relevance scale. Following content-validity index recommendations (Polit et al., 2007), item-level content validity indices ranged from 0.83 to 1.00, and the scale-level average content validity index was 0.96, supporting content validity. The items were then refined through a pilot survey with 180 undergraduate students. Pilot item analysis showed significant critical ratios for all items, item-total correlations ranging from 0.58 to 0.78, and subscale Cronbach’s alpha values ranging from 0.84 to 0.88. Exploratory factor analysis in the pilot sample supported the expected five-factor structure (KMO = 0.886; Bartlett’s chi-square = 1542.36, df = 105, *p* < 0.001; cumulative variance explained = 75.51%). Full content-validity, pilot item-analysis, and EFA results for the PE course-experience scale are reported in Supplementary Tables S2–S4. The ESE and PC measures were contextualized adaptations; their full contextualized English wording is provided in Appendix B, and independent psychometric validation remains necessary.

University PE course experience was measured as students’ perceived quality of engagement in PE courses, including supportive teaching, mastery-oriented learning, embodied participation, emotional release, and peer interaction. The scale included 15 items organized around five first-order dimensions: teacher support, mastery climate, embodied engagement, emotional release, and peer interaction. Because this construct was adapted to the university PE context, it was treated as a context-specific measure rather than as a fully established general scale. Its psychometric adequacy was therefore evaluated through internal consistency, convergent validity, discriminant validity, and second-order CFA before testing the structural model.

Resilience-based psychological recovery capacity was operationalized with the 10-item Connor-Davidson Resilience Scale (CD-RISC-10; Wang et al., 2010; Campbell-Sills and Stein, 2007). The administered 1–5 responses were averaged, with no reverse-scored items. Accordingly, the measured outcome reflects perceived adaptive capacity under sustained stress. It is not a direct measure of immediate relaxation, psychological detachment, control, or recovery following a specific PE lesson.

Emotion regulation was measured using the six-item cognitive reappraisal dimension of the Emotion Regulation Questionnaire (Wang et al., 2007; Gross and John, 2003). The administered 1–5 responses were averaged; none of the six reappraisal items was reverse scored. Cognitive reappraisal was selected because it reflects adaptive reinterpretation of stressful experiences and is theoretically aligned with resilience-based recovery.

Exercise self-efficacy refers to students’ confidence in their ability to persist in physical activity and manage exercise-related challenges. It was measured with six items adapted to the university PE context, focusing on confidence in completing PE tasks, maintaining exercise participation, and using physical activity to manage stress.

Peer connectedness refers to students’ sense of belonging, acceptance, and supportive interaction with classmates in PE courses. It was measured with six items referring specifically to classmates and peer interaction within PE courses rather than general friendship networks.

Perceived stress was measured using the 10-item Perceived Stress Scale (PSS-10), adapted from the Chinese validation reported by Lu et al. (2017) and retaining the original PSS construct. Items 4, 5, 7, and 8 were reverse scored using 6 minus the administered 1–5 response, and the 10 items were then averaged. The scale assesses the degree to which individuals perceive their lives as unpredictable, uncontrollable, and overloaded. In the SEM model, perceived stress was included as a covariate.

The primary covariates were perceived stress and extracurricular exercise frequency. Extracurricular exercise frequency was assessed with a single item asking how often students voluntarily exercised outside PE classes per week. Response categories were 0 times per week, 1 time per week, 2–3 times per week, and 4 or more times per week. Gender, grade level, and institution were recorded as sample descriptors. In the primary structural model, extracurricular exercise frequency was treated as an approximately continuous ordinal covariate, with a dummy-coded specification examined as a sensitivity analysis.

Because this item captures only voluntary exercise outside PE classes, it should not be interpreted as a measure of total physical-activity exposure or as isolating a causal PE-course effect. Duration, intensity, and total dose were not measured.

### 3.3 Data analysis

Data analysis proceeded in five steps. First, descriptive statistics, data-quality screening, and common method diagnostics were conducted. Participation was anonymous. Response-pattern exclusions were defined as the union of within-person raw-item SD < 0.20, cyclical responding across at least 15 consecutive items, or failure of either of the two attention checks. The separate completion-time criterion was <480 s. Exclusions were applied sequentially after eligibility screening to avoid double counting. Harman’s single-factor test was treated as a preliminary diagnostic. A 15-item PE common latent factor analysis evaluated within-scale loading sensitivity; neither diagnostic rules out common method variance across the study constructs (Podsakoff et al., 2003).

Data screening, descriptive statistics, reliability analysis, and preliminary correlation analyses were conducted using IBM SPSS Statistics 26.0. CFA and SEM were conducted in Mplus 8.3. Direct paths and model fit were estimated with robust maximum likelihood (MLR) using TYPE = COMPLEX and CLUSTER = teacher_id. The final SEM analyses were based on the complete valid analytic sample (*N* = 831) after eligibility, attention-check, and data-quality screening. Indirect associations and pairwise contrasts were evaluated in a complementary ML model using TYPE = COMPLEX, CLUSTER = teacher_id, and 5,000 bootstrap draws.

Second, reliability and validity were examined. Cronbach’s alpha and composite reliability were used to assess internal consistency. Confirmatory factor analysis evaluated convergent and discriminant validity. Average variance extracted, standardized factor loadings, the Fornell-Larcker criterion, and HTMT ratios were reported to reduce the risk that conceptually adjacent constructs were statistically indistinguishable (Fornell and Larcker, 1981; Henseler et al., 2015). Model fit was interpreted using multiple indices rather than relying on chi-square alone (Hu and Bentler, 1999; Kline, 2016).

Third, the structural model was tested using structural equation modeling. Model fit was evaluated using CFI, TLI, RMSEA, and SRMR. Direct associations among PE course experience, the three indirect-linking variables, and resilience-based psychological recovery capacity were examined.

Fourth, specific indirect associations and their pairwise differences were defined as path products and differences between products (Preacher and Hayes, 2008). Indirect associations involving emotion regulation, exercise self-efficacy, and peer connectedness were estimated with 5,000 teacher-cluster bootstrap draws under ML with TYPE = COMPLEX and CLUSTER = teacher_id. Bias-corrected bootstrap confidence intervals were obtained for the specific indirect associations, the total indirect association, and the pairwise contrasts. The primary direct paths and model fit used MLR with TYPE = COMPLEX and CLUSTER = teacher_id. Standardized (STDYX) point estimates are reported for the specific indirect associations, whereas MODEL CONSTRAINT pairwise contrasts are reported on the unstandardized indirect-effect scale.

Fifth, robustness checks examined a narrower PE predictor excluding Emotional Release and Peer Interaction and a dummy-coded extracurricular-exercise-frequency covariate. These analyses retained the three indirect-linking variables and adjusted for perceived stress. They evaluated sensitivity to predictor content and frequency coding, without establishing temporal or causal ordering.

## 4 Results

This section reports the results based on the current analytic dataset (*N* = 831). The analyses proceeded from data screening and sample description to measurement validation, common method bias assessment, structural path testing, and bootstrapped indirect-effect analysis.

### 4.1 Sample characteristics and data screening

A total of 900 questionnaires were distributed. Exclusions were mutually exclusive and sequential: 28 eligibility failures, 21 response-pattern quality failures, and 20 completion-time failures below 480 s, leaving 831 responses in the final analytic sample (92.3% of distributed questionnaires). The final sample included 398 male students and 433 female students, 455 undergraduate students and 376 vocational-college students. All items were forced-response items, so item-level missingness in the final analytic sample was zero. The four institutions contributed 253, 215, 202, and 161 respondents, respectively (Table 2).

**Table 2:** Sample characteristics and data screening.

Indicator	Value/*N*	Percentage (%)	Reporting note
Distributed questionnaires	900	100.0	Convenience and purposive sampling
Excluded: eligibility failure	28	3.1	Did not meet the PE-course eligibility criteria.
Excluded: response-pattern quality failures	21	2.3	Straight-lining, cyclical responding, or failed attention check.
Excluded: excessively short completion	20	2.2	Completion time <480 s.
Final analytic sample	831	92.3	Used for CFA and SEM.
Gender: male	398	47.9	
Gender: female	433	52.1	
Grade: freshman	335	40.3	Compulsory PE courses concentrated in the first 2 years.
Grade: sophomore	296	35.6	
Grade: junior	128	15.4	
Grade: senior	72	8.7	
Institution A	253	30.4	
Institution B	215	25.9	
Institution C	202	24.3	
Institution D	161	19.4	

### 4.2 Control variable coding and common method Bias

Extracurricular exercise frequency was an ordinal four-category variable and was treated as an approximately continuous covariate in the primary MLR specification as a pragmatic approximation. The category counts were 120 for 0 times per week, 215 for 1 time per week, 346 for 2–3 times per week, and 150 for 4 or more times per week. A dummy-coded sensitivity analysis used the 0-times-per-week group as the reference category. This coding should be interpreted as an adjustment for reported extracurricular exercise frequency, not as an exact measure of physical-activity dosage.

The 15-item PE common latent factor diagnostic used MLR. All 15 method-factor loadings were freely estimated, the method-factor variance was fixed at 1.0, and the method factor was specified as orthogonal to the substantive PE factors. The second-order baseline model yielded chi-square (85) = 215.50, CFI = 0.950, TLI = 0.939, RMSEA = 0.043 [0.037, 0.049], and SRMR = 0.041; the corresponding CLF model yielded chi-square (70) = 133.30, CFI = 0.976, TLI = 0.964, RMSEA = 0.033 [0.026, 0.040], and SRMR = 0.029. Standardized substantive-loading changes ranged from 0.012 to 0.054 (*M* = 0.027). This is a PE-scale sensitivity diagnostic and does not establish that common method variance is absent across the study constructs or that structural paths are unaffected. Supplementary Tables S8A,B provide the fit and loading comparisons.

### 4.3 Measurement model

PE Course Experience was modeled as a second-order latent construct comprising five first-order dimensions: teacher support, mastery climate, embodied engagement, emotional release, and peer interaction. The second-order loadings were strong for teacher support (0.82), mastery climate (0.79), embodied engagement (0.85), emotional release (0.81), and peer interaction (0.76). First-order item loadings were also acceptable across all dimensions. The second-order measurement model demonstrated good fit: chi-square (85) = 215.50, chi-square/df = 2.535, CFI = 0.950, TLI = 0.939, RMSEA = 0.043 [0.037, 0.049], and SRMR = 0.041. The specific item wording and the standardized first-order factor loadings for the 15 context-specific PE experience items are provided in Supplementary Table S1.

To evaluate the defensibility of this measurement specification, alternative CFA models were also compared. A single-factor model showed poor fit, indicating that PE course experience should not be treated as an undifferentiated perception. A correlated first-order five-factor model and the second-order five-factor model both demonstrated acceptable fit. The second-order model was retained for theoretical interpretability and parsimony rather than because it was uniquely superior to the alternative specifications. It represents overall course experience while preserving the five first-order dimensions. Full fit comparisons are reported in Supplementary Table S5. Bifactor and ESEM alternatives are summarized in Supplementary Table S6. These alternatives concern the internal representation of PE experience and do not establish its distinctiveness from unmeasured external constructs. Supplementary Table S7 reports scalar-invariance evidence for gender and educational sector (undergraduate versus vocational-college programs). S7 also reports scalar-invariance evidence across lower-division (first and second year; *N* = 631) and upper-division (third and fourth year; *N* = 200) students. Individual-institution invariance is not reported.

All constructs demonstrated satisfactory internal consistency and convergent validity. Cronbach’s alpha values ranged from 0.84 to 0.91, composite reliability values ranged from 0.85 to 0.90, and AVE values ranged from 0.53 to 0.65. Discriminant validity was supported by both the Fornell-Larcker criterion and HTMT ratios. All HTMT values were below the conservative threshold of 0.85. For PE Course Experience, Cronbach’s alpha was calculated across the 15 observed items, whereas CR and AVE for the higher-order construct were calculated from the five standardized second-order loadings (0.82, 0.79, 0.85, 0.81, and 0.76; Table 3).

**Table 3:** Reliability and convergent validity.

Construct	Items	Alpha	CR	AVE
Perceived PE course experience (2nd-order)	15	0.91	0.90	0.65
Emotion regulation (reappraisal)	6	0.84	0.85	0.58
Exercise self-efficacy	6	0.87	0.88	0.62
Peer connectedness	6	0.86	0.86	0.60
Resilience-based psychological recovery capacity	10	0.89	0.90	0.55
Perceived stress (control)	10	0.88	0.89	0.53

Table 4 shows that PE course experience was moderately correlated with resilience-based psychological recovery capacity and with the three proposed indirect-linking variables. The correlations were theoretically meaningful but not so high as to indicate construct redundancy. The negative correlation between perceived stress and recovery capacity further supports the criterion relevance of the recovery construct.

**Table 4:** Descriptive statistics, correlations, and HTMT ratios.

Variable	Mean	SD	1	2	3	4	5	6
1. PE Exp	3.85	0.72	(0.81)	[0.51]	[0.47]	[0.54]	[0.58]	[0.33]
2. Emotion Reg	3.62	0.81	0.45^***^	(0.76)	[0.43]	[0.40]	[0.52]	[0.37]
3. Ex Self-Eff	3.70	0.76	0.42^***^	0.38^***^	(0.79)	[0.46]	[0.49]	[0.29]
4. Peer Conn	3.91	0.74	0.48^***^	0.35^***^	0.41^***^	(0.77)	[0.47]	[0.26]
5. Recovery	3.58	0.68	0.51^***^	0.46^***^	0.44^***^	0.42^***^	(0.74)	[0.51]
6. Stress	3.12	0.85	−0.28^***^	−0.32^***^	−0.25^***^	−0.22^***^	−0.45^***^	(0.73)

Diagonal values in bold parentheses are square roots of AVE. Values below the diagonal are Pearson correlations. Values above the diagonal in brackets [] are Heterotrait-Monotrait (HTMT) ratios. All off-diagonal correlations were below the corresponding diagonal values, and all HTMT ratios were below the conservative threshold of 0.85, providing evidence of discriminant validity within the measured construct set. ^***^p < 0.001.

The discriminant-validity statistics do not fully eliminate item-content overlap. Emotional Release and Peer Interaction are components of the PE course experience construct, while emotion regulation and peer connectedness are modeled as separate indirect-linking variables. The constructs differ in level and referent: the PE dimensions evaluate perceived features or consequences of a specific course context, whereas the indirect-linking variables represent broader personal and relational resources. Nevertheless, similarly worded items may inflate their observed associations. The findings should therefore be interpreted alongside the narrow-PE sensitivity specification that excludes Emotional Release and Peer Interaction, rather than relying on HTMT and factor separation alone.

### 4.4 Structural model and indirect associations

The structural model demonstrated acceptable fit to the data: chi-square/df = 2.18, CFI = 0.94, TLI = 0.93, RMSEA = 0.045, and SRMR = 0.042. PE Course Experience was positively associated with Resilience-Based Psychological Recovery Capacity (beta = 0.24, SE = 0.055, 95% CI [0.13, 0.35], *p* < 0.001), Emotion Regulation (beta = 0.41, SE = 0.042, 95% CI [0.33, 0.49], *p* < 0.001), Exercise Self-Efficacy (beta = 0.39, SE = 0.045, 95% CI [0.30, 0.48], *p* < 0.001), and Peer Connectedness (beta = 0.46, SE = 0.040, 95% CI [0.38, 0.54], *p* < 0.001). The Peer Connectedness-to-Recovery path was positive (beta = 0.15, SE = 0.050, 95% CI [0.05, 0.25], *p* = 0.003).

The model explained variance in the endogenous variables. PE course experience explained 17% of the variance in emotion regulation, 15% of the variance in exercise self-efficacy, and 21% of the variance in peer connectedness. The full structural model explained 39% of the variance in resilience-based psychological recovery capacity. These R-squared values indicate moderate explanatory power for the proposed indirect-association model (Tables 5, 6).

**Table 5:** Direct associations in the structural equation model.

Hypothesis/path	Structural path	Std. estimate	SE	95% CI	*p*	Conclusion
H1	PE experience → Psychological recovery	0.24	0.055	[0.13, 0.35]	<0.001	Supported
H2	PE experience → Emotion regulation	0.41	0.042	[0.33, 0.49]	<0.001	Supported
H3	PE experience → Exercise self-efficacy	0.39	0.045	[0.30, 0.48]	<0.001	Supported
H4	PE experience → Peer connectedness	0.46	0.040	[0.38, 0.54]	<0.001	Supported
Structural path	Emotion regulation → Psychological recovery	0.20	0.051	[0.10, 0.30]	<0.001	Reported
Structural path	Exercise self-efficacy → Psychological recovery	0.26	0.048	[0.17, 0.35]	<0.001	Reported
Structural path	Peer connectedness → Psychological recovery	0.15	0.050	[0.05, 0.25]	0.003	Reported
Covariate	Perceived stress → Psychological recovery	−0.36	0.036	[−0.43, −0.29]	<0.001	Significant
Covariate	Extracurricular exercise frequency → Psychological recovery	0.11	0.045	[0.02, 0.20]	0.014	Significant

**Table 6:** Parallel indirect associations using 5,000 bootstrap samples.

Hypothesis	Indirect path	Estimate (STDYX)	Boot SE	LLCI	ULCI	Conclusion
H5	PE exp → Emotion regulation → Recovery	0.08	0.021	0.05	0.13	Supported
H6	PE exp → Exercise self-efficacy → Recovery	0.10	0.022	0.07	0.15	Supported
H7	PE exp → Peer connectedness → Recovery	0.07	0.020	0.04	0.12	Supported
Total indirect	Sum of the three indirect paths	0.25	0.031	0.20	0.32	Supported

Standardized specific indirect point estimates and the total indirect association were obtained from the complementary ML model using TYPE = COMPLEX, CLUSTER = teacher_id, and 5,000 bootstrap draws. LLCI and ULCI denote the lower and upper limits of the reported 95% confidence intervals. The primary direct-path estimate is reported separately in Table 5 from the MLR model.

The indirect association through emotion regulation was 0.08 (95% CI [0.05, 0.13]), through exercise self-efficacy was 0.10 (95% CI [0.07, 0.15]), and through peer connectedness was 0.07 (95% CI [0.04, 0.12]). The total indirect association was 0.25 (95% CI [0.20, 0.32]). Pairwise contrasts on the unstandardized scale further revealed that the indirect association via exercise self-efficacy was significantly larger than that via peer connectedness (difference = 0.03, 95% CI [0.006, 0.054]), whereas the differences between exercise self-efficacy and emotion regulation (difference = 0.02, 95% CI [−0.002, 0.042]) and between emotion regulation and peer connectedness (difference = 0.01, 95% CI [−0.010, 0.030]) were not statistically significant. In the primary MLR model, the direct association between PE course experience and resilience-based psychological recovery capacity was 0.24 (95% CI [0.13, 0.35]), and the total association was 0.49 (SE = 0.032, 95% CI [0.43, 0.55], *p* < 0.001; Figure 2).

**Figure 2:**
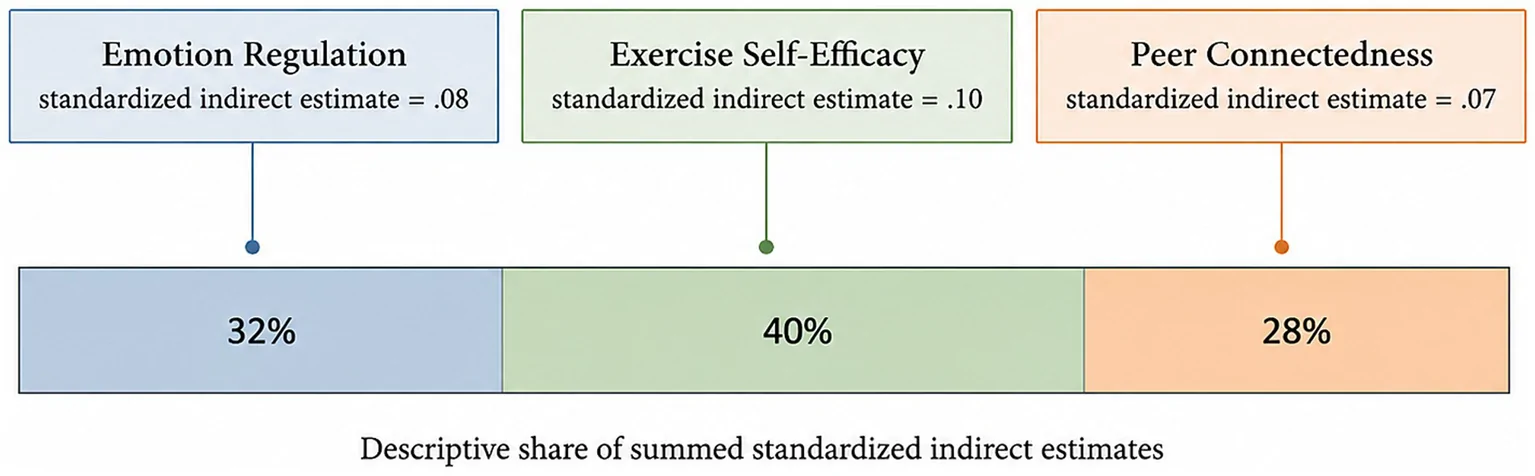
Descriptive shares of the reported total indirect association. Shares summarize rounded point estimates and do not establish pairwise statistical differences; formal contrasts are reported in Supplementary Table S10.

### 4.5 Supplementary robustness checks

Several supplementary analyses were conducted to examine whether the reported associations were robust to plausible measurement and coding alternatives. These included removal of the Emotional Release and Peer Interaction dimensions from the PE predictor and dummy-coding extracurricular exercise frequency. The results were treated as robustness evidence, not as tests of temporal ordering or causal effects (Table 7).

**Table 7:** Supplementary robustness checks.

Check	Model or criterion	Key result	Interpretation
Narrow-PE sensitivity	Remove ERL and PI from PE predictor	PE → ER 0.39; PE → ESE 0.38; PE → PC 0.44; PE → Recovery 0.23	Direction and magnitude stable
Indirect sensitivity	Narrow PE predictor	ER 0.07; ESE 0.10; PC 0.06	Direction preserved; no causal claim
Extracurricular exercise frequency	Dummy-coded; 0-times-per-week group as reference	0.09, 0.12, 0.10 for the three higher-frequency groups	Focal paths stable
Pairwise indirect contrasts	5,000 bootstrap samples	ESE-PC 0.03 significant; ESE-ER 0.02 and ER-PC 0.01 not significant	ESE is largest point estimate, not statistically superior to ER

The supplementary checks are reported only as robustness comparisons. Because the data are cross-sectional, relative fit or stable estimates do not establish temporal or causal ordering.

## 5 Discussion

H1–H4 were supported by positive direct associations between perceived PE course experience and resilience-based psychological recovery capacity, emotion regulation, exercise self-efficacy, and peer connectedness. H5–H7 were supported because the three proposed specific indirect associations had bias-corrected teacher-cluster bootstrap intervals that excluded zero. Exercise self-efficacy had the largest point estimate, and its indirect association was statistically larger than the peer-connectedness association, but it was not statistically distinguishable from the emotion-regulation association. These findings support a bounded claim: perceived university PE course experience is associated with resilience-based psychological recovery capacity through several proposed resource pathways; they do not establish temporal or causal mechanisms.

### 5.1 PE course experience as a multidimensional course ecology

The H1 association extends prior exercise and mental-health research in a specifically educational direction. Existing reviews and meta-analyses have established that physical activity is associated with lower depression, anxiety, and psychological distress. The present study does not simply repeat that conclusion. Instead, it examines whether students’ perceived experience of PE courses is associated with resilience-based psychological recovery capacity after accounting for extracurricular exercise frequency. This is an incremental conditional association, not evidence of a uniquely institutional or causal effect.

The second-order measurement result further strengthens this interpretation. Teacher support, mastery climate, embodied engagement, emotional release, and peer interaction all loaded strongly onto the higher-order PE experience factor. This suggests that PE is experienced by students as a course ecology rather than a single behavioral exposure. The value of PE therefore lies not only in movement itself, but in how movement is pedagogically structured, socially organized, and affectively experienced. This is where the present study moves beyond the conventional exercise-as-behavior framework and positions PE as a recovery-supportive course context.

It is also important to clarify the meaning of embodiment in the present study. The data do not measure objective physiological embodiment, such as movement intensity, heart rate, or accelerometer-based activity. Rather, they capture perceived embodied experience: students’ subjective sense that PE helps them shift attention away from academic pressure, reconnect with bodily states, feel energized, and experience emotional release. This boundary is theoretically appropriate for an educational psychology study because the question is not only whether bodies move, but how students interpret and give psychological meaning to movement within a structured course context.

### 5.2 Exercise self-efficacy as the largest estimated indirect association

Exercise self-efficacy had the largest standardized indirect point estimate (0.10), followed by emotion regulation (0.08) and peer connectedness (0.07). On the unstandardized indirect-association scale, teacher-cluster bootstrap contrasts supported a larger association through ESE than through PC (difference = 0.03, 95% CI [0.006, 0.054]). ESE versus ER (0.02, 95% CI [−0.002, 0.042]) and ER versus PC (0.01, 95% CI [−0.010, 0.030]) included zero. Thus, the contrast evidence supports one pairwise difference and does not establish that ESE exceeds both alternatives.

This pattern is compatible with the competence component of self-determination theory (Ryan and Deci, 2000). Mastery-oriented course experiences may be relevant to perceived capability, but the present design does not directly test the development of competence. The absence of a statistically distinguishable ESE-ER contrast also cautions against prioritizing self-efficacy over emotion regulation solely on the basis of these estimates.

### 5.3 Emotion regulation and peer connectedness as complementary recovery pathways

Emotion regulation also showed a significant indirect association, supporting H2 and H5. This result aligns with Gross's (1998) account of adaptive functioning and can be interpreted alongside recovery theory, which emphasizes relaxation, mastery, control, and detachment as pathways for restoring depleted resources (Sonnentag and Fritz, 2007). The present model does not test whether physiological activation enables cognitive reappraisal. Rather, it identifies a cross-sectional association between perceived PE course experience and a self-reported emotion-regulation resource. The proposed bridge between bodily participation and cognitive reappraisal remains a question for repeated-measure and behavioral studies.

Peer connectedness supported H4 and H7 and adds a social layer to the recovery argument. This result is compatible with the stress-buffering model of social support (Cohen and Wills, 1985), but the present data do not establish stress buffering or changes in social connection over time. Cooperative course activities provide a relevant context for future intervention research. Whether such contexts reduce perceived stigma or reach students who avoid formal help-seeking requires direct measurement rather than inference from the present associations.

### 5.4 PE course experience and extracurricular exercise frequency

The PE-course association remained positive after adjustment for extracurricular exercise frequency. This should be interpreted as an incremental conditional association within the present specification, not as proof that PE-course experience has a uniquely institutional or causal effect. The exercise-frequency item captured only the reported weekly frequency of voluntary exercise outside PE classes and did not measure duration, intensity, or total physical-activity dose.

This interpretation may be relevant beyond the Chinese setting because many universities face a mismatch between student distress and the reach of formal mental health services. The present study did not directly measure stigma, scalability, service reach, or institutional implementation. These ideas are therefore presented as theoretically grounded implications for curriculum design, not as empirically demonstrated properties of the participating PE programs.

The supplementary robustness checks were narrower. Removing Emotional Release and Peer Interaction from the PE predictor left the focal paths in the same direction: PE-to-ER 0.39, PE-to-ESE 0.38, PE-to-PC 0.44, and direct PE-to-Recovery 0.23; the indirect associations were approximately 0.07, 0.10, and 0.06. Dummy-coding extracurricular exercise frequency with the 0-times-per-week group as the reference produced coefficients of 0.09, 0.12, and 0.10 for the three higher-frequency groups. These checks support stability of the reported associations, not causal ordering.

### 5.5 Theoretical and practical implications

At the theoretical level, the findings link perceived PE course experience with resilience-based psychological recovery capacity in a model that includes emotion regulation, exercise self-efficacy, and peer connectedness as statistically linked resource variables. This supports a course-ecology framing in which teacher support, mastery orientation, embodied participation, emotional experience, and peer interaction are considered jointly. It does not demonstrate that PE produces recovery, that the proposed variables operate as temporal mechanisms, or that the course setting is distinct from the measured constructs in external validation.

These associations warrant prospective evaluation of specific course practices rather than immediate policy conclusions. Intervention or implementation studies could test whether progress-oriented framing, supportive feedback, and cooperative tasks change students’ perceived PE experience or recovery-related outcomes. The present study did not evaluate effects, cost, implementation fidelity, reach, stigma, or unintended consequences, so these practices should be considered testable options that complement, rather than replace, professional mental-health services.

Any translation into practice should preserve the distinction between educational support and clinical care. PE teachers should not be positioned as substitute counselors and should retain appropriate referral pathways. Whether course redesign changes recovery-related outcomes remains a question for prospective research.

### 5.6 Limitations and future research

The cross-sectional design leaves temporal ordering and reverse associations unresolved. Students with greater resilience may evaluate PE more positively, and unmeasured course or personal characteristics may influence both. All focal measures were self-reported. Although the 15-item PE method-factor sensitivity analysis showed small changes in substantive loadings, common method variance may still inflate the observed associations. No marker-variable measure or multi-source validation subsample was available. The 15-item CLF specification and item-level loading comparison are documented in Supplementary Tables S8A,B and Appendix D. Teacher-cluster inference addresses dependence within teachers but does not resolve these design limitations. Longitudinal and multi-method replication is needed.

Resilience-based psychological recovery capacity was operationalized through resilience. Although this is theoretically defensible, resilience is not identical to momentary recovery. Future research should incorporate direct recovery experience measures, such as relaxation, mastery, control, and psychological detachment, and should examine whether PE course experiences are associated with changes in these states over time. The PE course experience measure was developed through expert review, pilot testing, and psychometric evaluation in the present sample, but it remains a context-specific instrument requiring independent cross-validation. Independent measures of autonomy support (e.g., PASSES), mastery climate, enjoyment, physical self-concept, and exercise motivation were not administered. The literature-based distinctions in referent and scope cannot substitute for concurrent external validation. Alternative measurement models, HTMT ratios, and the narrow-PE analysis provide evidence about internal structure and sensitivity within the measured construct set; they do not establish external discriminant validity or incremental value over established instruments. Future independent samples should include these instruments concurrently, assess their latent associations and content overlap, and test whether the broader PE measure adds explanatory or prospective predictive value. Scalar-invariance results concern gender, educational sector, and lower- versus upper-division grade stage. They do not establish invariance across individual grades or individual institutions. Future studies should combine independent validation samples, external measurement batteries, and prespecified group comparisons.

In addition, the extracurricular exercise-frequency covariate captured only the reported weekly frequency of voluntary exercise outside PE classes. It did not measure exercise duration, intensity, or total physical-activity dose, nor did it quantify the amount or intensity of physical activity occurring within PE classes. The analysis therefore provides only a coarse adjustment for extracurricular exercise behavior rather than comprehensive control of total physical-activity exposure. Future research should use validated physical-activity instruments or objective measures such as accelerometers to distinguish frequency, duration, and intensity more precisely.

### 5.7 Conclusion

Positive perceived university PE course experience was associated with stronger resilience-based psychological recovery capacity among the surveyed students. Indirect associations were also observed through emotion regulation, exercise self-efficacy, and peer connectedness. These cross-sectional estimates support further study of PE as a potentially recovery-supportive educational context; they do not establish temporal direction, causal effects, stigma reduction, scalability, or implementation impact.

## Data Availability

The datasets generated and/or analyzed during the current study are not publicly available because they contain student survey responses and institutional information. De-identified data may be made available from the corresponding author, Meng Liangfang, upon reasonable request and with appropriate ethical approval. Requests to access the datasets should be directed to mlf19881@outlook.com.
