# Supplementary material for: University physical education course experience and resilience-based psychological recovery capacity among students in Chinese higher education: indirect associations through emotion regulation, exercise self-efficacy, and peer connectedness
Source: Front Psychol. 2026 Oct 15;17:1908934. doi: 10.3389/fpsyg.2026.1908934 (PMC13716775; doi:10.3389/fpsyg.2026.1908934)
Supplement: Supplementary file 1 [file Supplementary_file_1.DOCX]

**Supplementary Material**

# Appendix A (Supplementary Table S1). PE Course Experience Scale Items and First-Order Factor Loadings

English translations of the item wording for the Perceived PE Course Experience scale. Items were originally developed in Chinese, translated into English for reporting, and rated on a 5-point Likert-type scale from 1 = strongly disagree to 5 = strongly agree.

| **Dimension** | **Item Code** | **English Item Wording (translated from Chinese)** | **Baseline Std. Loading** |
| --- | --- | --- | --- |
| Teacher Support | TS1 | In PE class, the teacher creates a classroom atmosphere where students feel respected. | 0.692 |
| Teacher Support | TS2 | The PE teacher encourages students to evaluate their performance based on personal progress rather than just comparing with others. | 0.755 |
| Teacher Support | TS3 | The feedback from the PE teacher makes me feel capable of continuing to improve. | 0.810 |
| Mastery Climate | MC1 | PE class gives me the opportunity to experience my own progress through practice. | 0.675 |
| Mastery Climate | MC2 | PE class makes me believe that diligent practice can lead to improvement. | 0.742 |
| Mastery Climate | MC3 | This PE course emphasizes learning and personal development, rather than just grades or test results. | 0.801 |
| Embodied Engagement | EE1 | During PE class, I can shift my attention from academic pressure to physical activity. | 0.720 |
| Embodied Engagement | EE2 | After long periods of studying or using electronic screens, PE class gives me the opportunity to reconnect with my bodily state. | 0.788 |
| Embodied Engagement | EE3 | The physical activities in PE class make me feel more energized. | 0.851 |
| Emotional Release | ERL1 | PE class provides a useful way for me to release tension. | 0.657 |
| Emotional Release | ERL2 | After participating in PE class, I usually feel emotionally lighter. | 0.738 |
| Emotional Release | ERL3 | PE activities help me recover from negative emotions accumulated during study or daily life. | 0.825 |
| Peer Interaction | PI1 | PE class gives me the opportunity to interact naturally with classmates. | 0.713 |
| Peer Interaction | PI2 | Group or cooperative activities in PE class make me feel more connected with other classmates. | 0.787 |
| Peer Interaction | PI3 | Cooperating with peers in PE class makes the course experience feel more supportive. | 0.854 |

*Note. N = 831. The table reports the baseline standardized first-order loadings for the 15 PE course-experience items. Item-level SEs and p values were not available in the re-estimation summary and are therefore not inferred here. The five first-order dimensions loaded onto the higher-order PE factor with standardized loadings of .82, .79, .85, .81, and .76, respectively.*

# Supplementary Table S2. Expert Content Validity Ratings for PE Course Experience Items

Six experts with backgrounds in physical education pedagogy and psychometrics rated each item on a 4-point relevance scale from 1 = not relevant to 4 = highly relevant. I-CVI represents the proportion of experts assigning a rating of 3 or 4.

| **Dimension** | **Item** | **E1** | **E2** | **E3** | **E4** | **E5** | **E6** | **Experts 3/4** | **I-CVI** |
| --- | --- | --- | --- | --- | --- | --- | --- | --- | --- |
| Teacher Support | PE1 | 4 | 4 | 4 | 3 | 4 | 4 | 6 | 1.00 |
| Teacher Support | PE2 | 4 | 4 | 3 | 4 | 4 | 4 | 6 | 1.00 |
| Teacher Support | PE3 | 4 | 3 | 4 | 4 | 2 | 4 | 5 | 0.83 |
| Mastery Climate | PE4 | 4 | 4 | 4 | 4 | 4 | 3 | 6 | 1.00 |
| Mastery Climate | PE5 | 4 | 4 | 4 | 4 | 4 | 4 | 6 | 1.00 |
| Mastery Climate | PE6 | 3 | 4 | 2 | 4 | 4 | 4 | 5 | 0.83 |
| Embodied Engagement | PE7 | 4 | 4 | 4 | 3 | 4 | 4 | 6 | 1.00 |
| Embodied Engagement | PE8 | 4 | 4 | 4 | 4 | 4 | 4 | 6 | 1.00 |
| Embodied Engagement | PE9 | 4 | 4 | 4 | 4 | 4 | 4 | 6 | 1.00 |
| Emotional Release | PE10 | 4 | 3 | 4 | 4 | 2 | 4 | 5 | 0.83 |
| Emotional Release | PE11 | 4 | 4 | 4 | 3 | 4 | 4 | 6 | 1.00 |
| Emotional Release | PE12 | 4 | 4 | 4 | 4 | 4 | 4 | 6 | 1.00 |
| Peer Interaction | PE13 | 4 | 4 | 4 | 4 | 4 | 4 | 6 | 1.00 |
| Peer Interaction | PE14 | 2 | 4 | 4 | 4 | 4 | 3 | 5 | 0.83 |
| Peer Interaction | PE15 | 4 | 4 | 4 | 3 | 4 | 4 | 6 | 1.00 |

*Note. S-CVI/Ave = .96, calculated from the unrounded item proportions: (11 + 4 x 5/6)/15 = .9556. Eleven items received relevance ratings of 3 or 4 from all six experts, and four received such ratings from five experts (Polit et al., 2007).*

# Supplementary Table S3. Pilot Item Analysis and Internal Consistency of the PE Course Experience Scale

The pilot survey included 180 undergraduate students. Critical ratios were based on independent-samples t-tests comparing the upper 27% and lower 27% scoring groups.

| **Dimension / Item** | **Mean** | **SD** | **Skewness** | **Kurtosis** | **CR** | **ITC** | **Alpha if deleted** |
| --- | --- | --- | --- | --- | --- | --- | --- |
| Teacher Support (alpha = 0.86) |  |  |  |  |  |  |  |
| PE1 | 3.82 | 0.74 | -0.42 | 0.15 | 11.24*** | 0.68 | 0.81 |
| PE2 | 3.75 | 0.81 | -0.35 | -0.22 | 10.58*** | 0.65 | 0.82 |
| PE3 | 3.88 | 0.76 | -0.51 | 0.34 | 12.07*** | 0.72 | 0.78 |
| Mastery Climate (alpha = 0.84) |  |  |  |  |  |  |  |
| PE4 | 3.65 | 0.85 | -0.28 | -0.45 | 9.85*** | 0.62 | 0.81 |
| PE5 | 3.71 | 0.79 | -0.31 | -0.18 | 10.42*** | 0.67 | 0.78 |
| PE6 | 3.58 | 0.88 | -0.15 | -0.51 | 9.12*** | 0.58 | 0.82 |
| Embodied Engagement (alpha = 0.88) |  |  |  |  |  |  |  |
| PE7 | 3.92 | 0.72 | -0.62 | 0.45 | 13.15*** | 0.75 | 0.82 |
| PE8 | 3.85 | 0.75 | -0.48 | 0.21 | 12.64*** | 0.71 | 0.84 |
| PE9 | 3.96 | 0.68 | -0.68 | 0.62 | 13.88*** | 0.78 | 0.80 |
| Emotional Release (alpha = 0.85) |  |  |  |  |  |  |  |
| PE10 | 3.78 | 0.82 | -0.38 | -0.12 | 10.92*** | 0.66 | 0.81 |
| PE11 | 3.64 | 0.86 | -0.25 | -0.33 | 9.76*** | 0.61 | 0.84 |
| PE12 | 3.81 | 0.79 | -0.41 | -0.05 | 11.45*** | 0.69 | 0.79 |
| Peer Interaction (alpha = 0.87) |  |  |  |  |  |  |  |
| PE13 | 4.02 | 0.65 | -0.75 | 0.81 | 14.52*** | 0.74 | 0.81 |
| PE14 | 3.89 | 0.73 | -0.55 | 0.28 | 12.33*** | 0.68 | 0.84 |
| PE15 | 3.95 | 0.70 | -0.61 | 0.52 | 13.08*** | 0.72 | 0.82 |

*Note. N = 180. *** p < .001. CR = critical ratio; ITC = item-total correlation.*

# Supplementary Table S4. Exploratory Factor Analysis Pattern Matrix for the PE Course Experience Scale

Exploratory factor analysis used principal axis factoring with Promax rotation. KMO = 0.886; Bartlett's test of sphericity: chi-square = 1542.36, df = 105, p < .001.

| **Item** | **Factor 1 (EE)** | **Factor 2 (PI)** | **Factor 3 (TS)** | **Factor 4 (ER)** | **Factor 5 (MC)** | **h2** |
| --- | --- | --- | --- | --- | --- | --- |
| PE7 | 0.86 | 0.05 | -0.02 | 0.12 | 0.04 | 0.68 |
| PE8 | 0.82 | 0.08 | 0.06 | -0.05 | 0.11 | 0.65 |
| PE9 | 0.89 | -0.03 | 0.04 | 0.08 | -0.06 | 0.71 |
| PE13 | 0.06 | 0.84 | 0.11 | -0.02 | 0.07 | 0.67 |
| PE15 | -0.04 | 0.81 | 0.05 | 0.14 | -0.03 | 0.64 |
| PE14 | 0.09 | 0.77 | -0.08 | 0.06 | 0.12 | 0.61 |
| PE3 | -0.02 | 0.04 | 0.85 | 0.09 | 0.05 | 0.66 |
| PE1 | 0.11 | -0.06 | 0.82 | -0.04 | 0.14 | 0.63 |
| PE2 | 0.05 | 0.12 | 0.78 | 0.07 | -0.08 | 0.59 |
| PE10 | 0.14 | -0.02 | 0.05 | 0.83 | 0.06 | 0.65 |
| PE12 | -0.06 | 0.08 | -0.03 | 0.80 | 0.15 | 0.62 |
| PE11 | 0.08 | 0.05 | 0.12 | 0.76 | -0.04 | 0.58 |
| PE4 | 0.03 | 0.11 | -0.05 | 0.08 | 0.81 | 0.61 |
| PE6 | -0.05 | -0.04 | 0.15 | -0.02 | 0.78 | 0.58 |
| PE5 | 0.12 | 0.06 | 0.08 | 0.05 | 0.75 | 0.56 |
| Eigenvalues | 5.82 | 1.85 | 1.42 | 1.18 | 1.06 |  |
| % of variance | 38.80 | 12.33 | 9.46 | 7.86 | 7.06 |  |
| Cumulative % | 38.80 | 51.13 | 60.59 | 68.45 | 75.51 |  |

*Note. Extraction method: Principal Axis Factoring. Rotation method: Promax with Kaiser normalization. EE = Embodied Engagement; PI = Peer Interaction; TS = Teacher Support; ER = Emotional Release; MC = Mastery Climate. The communalities sum to 9.44, corresponding to 62.93% when expressed over 15 standardized indicators. The cumulative 75.51% is the cumulative initial eigenvalue-based variance reported by the extraction output and is not treated as the communality-based proportion.*

# Supplementary Table S5. Alternative Measurement Model Comparisons for the PE Course Experience Scale

Alternative CFA models were compared to evaluate whether the proposed five-dimensional structure was preferable to a single undifferentiated factor and whether a higher-order representation was theoretically and statistically defensible.

| **Model** | **Specification** | **chi-square** | **df** | **chi-square/df** | **CFI** | **TLI** | **RMSEA [90% CI]** | **SRMR** |
| --- | --- | --- | --- | --- | --- | --- | --- | --- |
| Single-factor model | All 15 items loaded onto one PE experience factor | 685.80 | 90 | 7.62 | .71 | .66 | .089 [.083, .095] | .086 |
| Correlated first-order five-factor model | Five first-order factors were freely correlated | 168.72 | 80 | 2.11 | .96 | .95 | .040 [.032, .048] | .038 |
| Second-order five-factor model | Five first-order factors loaded onto one higher-order PE experience factor | 215.50 | 85 | 2.535 | .950 | .939 | .043 [.037, .049] | .041 |

*Note. The single-factor model showed poor fit, supporting the multidimensionality of PE course experience. The correlated first-order model and second-order model both demonstrated acceptable fit. The second-order model was retained because it provided a theoretically parsimonious representation of PE course experience as an overarching course ecology while preserving the five first-order dimensions.*

# Supplementary Table S6. Bifactor and ESEM Alternative Measurement Models

| **Model** | **chi-square** | **df** | **chi-square/df** | **CFI** | **TLI** | **RMSEA [90% CI]** | **SRMR** | **Diagnostic conclusion** |
| --- | --- | --- | --- | --- | --- | --- | --- | --- |
| Second-order five-factor model | 215.50 | 85 | 2.535 | .950 | .939 | .043 [.037, .049] | .041 | Retained benchmark model |
| Bifactor model | 134.12 | 70 | 1.92 | .97 | .96 | .038 [.029, .046] | .034 | General and specific factors estimated; no Heywood cases |
| Five-factor ESEM | 122.45 | 55 | 2.23 | .97 | .95 | .044 [.034, .054] | .031 | Target-rotation primary loadings >.55; cross-loadings <.20 |

Note. The second-order model was retained on theoretical and parsimony grounds; bifactor and ESEM specifications were examined as alternative representations of the PE measure. Non-distinct-model chi-square comparisons are not reported because matched joint baseline models with identical indicator sets were not documented. Evidence concerning overlap with the other measured constructs is reported through HTMT ratios (main Table 4) and the narrow-PE sensitivity analysis (Supplementary Table S11). These checks do not replace validation against independent external measures.

# Supplementary Table S7. Measurement Invariance of the PE Course Experience Scale

| **Grouping variable** | **Model** | **chi-square** | **df** | **CFI** | **TLI** | **RMSEA** | **SRMR** | **Delta CFI** | **Delta RMSEA** | **Delta SRMR** | **Decision** |
| --- | --- | --- | --- | --- | --- | --- | --- | --- | --- | --- | --- |
| Gender (398 male, 433 female) | Configural | 325.40 | 160 | .952 | .941 | .041 | .039 | — | — | — | Baseline |
| Gender (398 male, 433 female) | Metric | 334.12 | 170 | .951 | .944 | .040 | .041 | -.001 | -.001 | .002 | Supported |
| Gender (398 male, 433 female) | Scalar | 346.80 | 180 | .947 | .943 | .040 | .043 | -.004 | .000 | .002 | Supported |
| Educational sector (455 undergraduate / 376 vocational) | Configural | 339.15 | 160 | .949 | .938 | .043 | .041 | — | — | — | Baseline |
| Educational sector (455 undergraduate / 376 vocational) | Metric | 348.90 | 170 | .947 | .941 | .042 | .043 | -.002 | -.001 | .002 | Supported |
| Educational sector (455 undergraduate / 376 vocational) | Scalar | 361.20 | 180 | .943 | .940 | .042 | .045 | -.004 | .000 | .002 | Supported |
| Grade stage (631 lower / 200 upper) | Configural | 345.80 | 160 | .947 | .936 | .043 | .041 | — | — | — | Baseline |
| Grade stage (631 lower / 200 upper) | Metric | 356.10 | 170 | .945 | .940 | .042 | .043 | -.002 | -.001 | .002 | Supported |
| Grade stage (631 lower / 200 upper) | Scalar | 369.40 | 180 | .941 | .938 | .042 | .045 | -.004 | .000 | .002 | Supported |

Note. Configural, metric, and scalar models were examined for gender, educational sector (undergraduate versus vocational-college programs), and grade stage (lower division, N = 631, versus upper division, N = 200). Sequential changes in CFI and RMSEA were consistent with scalar invariance using criteria of a CFI decrease no greater than .010 and an RMSEA increase no greater than .015. These results do not establish strict residual invariance, invariance across individual grades, or invariance across individual institutions.

# Supplementary Table S8A. Common Latent Factor Model Fit for the 15 PE Items

| **Model specification** | **chi-square** | **df** | **chi-square/df** | **CFI** | **TLI** | **RMSEA [90% CI]** | **SRMR** |
| --- | --- | --- | --- | --- | --- | --- | --- |
| 15-item second-order baseline CFA (without CLF) | 215.50 | 85 | 2.535 | .950 | .939 | .043 [.037, .049] | .041 |
| 15-item CLF model | 133.30 | 70 | 1.904 | .976 | .964 | .033 [.026, .040] | .029 |

Note. N = 831. Both models concern only the 15 PE course-experience indicators. The baseline is the second-order PE measurement model; the CLF model adds one orthogonal common method factor with all 15 method-factor loadings freely estimated and the method-factor variance fixed at 1.0. The net degrees-of-freedom difference is 15. Fit indices are reported from the re-estimated models and are interpreted as a sensitivity comparison, not as evidence that common method bias is absent.

Supplementary Table S8B. Summary of Substantive Loading Changes for the 15 PE Items

| **Item Code** | **Baseline Loading** | **Post-CLF Loading** | **Absolute Change** | **CLF Loading** | **Method Variance (gamma^2)** |
| --- | --- | --- | --- | --- | --- |
| TS1 | .692 | .680 | .012 | .128 | 1.64% |
| TS2 | .755 | .740 | .015 | .150 | 2.25% |
| TS3 | .810 | .790 | .020 | .180 | 3.24% |
| MC1 | .675 | .660 | .015 | .142 | 2.02% |
| MC2 | .742 | .720 | .022 | .180 | 3.24% |
| MC3 | .801 | .770 | .031 | .220 | 4.84% |
| EE1 | .720 | .700 | .020 | .170 | 2.89% |
| EE2 | .788 | .760 | .028 | .210 | 4.41% |
| EE3 | .851 | .810 | .041 | .260 | 6.76% |
| ERL1 | .657 | .640 | .017 | .150 | 2.25% |
| ERL2 | .738 | .710 | .028 | .200 | 4.00% |
| ERL3 | .825 | .780 | .045 | .270 | 7.29% |
| PI1 | .713 | .690 | .023 | .180 | 3.24% |
| PI2 | .787 | .750 | .037 | .240 | 5.76% |
| PI3 | .854 | .800 | .054 | .300 | 9.00% |

Note. Standardized estimates are reported at the available precision. Absolute substantive-loading changes ranged from .012 to .054 (M = .027). CLF loadings ranged from .128 to .300 (M = .199), and the mean method-variance proportion was 4.19%. Within this specified PE measurement model, the results suggest limited sensitivity of substantive item loadings to the added method factor; they do not establish that common method variance is absent across the study constructs or that structural paths are unaffected.

# Supplementary Table S9. Full Structural Model Results

| **Path/effect** | **Standardized estimate** | **SE** | **95% CI** | **p** | **Status** |
| --- | --- | --- | --- | --- | --- |
| PE experience -> emotion regulation (a1) | .41 | .042 | [.33, .49] | < .001 | Reported |
| PE experience -> exercise self-efficacy (a2) | .39 | .045 | [.30, .48] | < .001 | Reported |
| PE experience -> peer connectedness (a3) | .46 | .040 | [.38, .54] | < .001 | Reported |
| Emotion regulation -> resilience-based recovery capacity (b1) | .20 | .051 | [.10, .30] | < .001 | Estimated |
| Exercise self-efficacy -> resilience-based recovery capacity (b2) | .26 | .048 | [.17, .35] | < .001 | Estimated |
| Peer connectedness -> resilience-based recovery capacity (b3) | .15 | .050 | [.05, .25] | .003 | Estimated |
| PE experience -> resilience-based recovery capacity (c') | .24 | .055 | [.13, .35] | < .001 | Reported |
| Perceived stress -> resilience-based recovery capacity | -.36 | .036 | [-.43, -.29] | < .001 | Reported |
| Extracurricular exercise frequency -> resilience-based recovery capacity | .11 | .045 | [.02, .20] | .014 | Reported |
| Specific indirect: emotion regulation | .08 | .021 | [.05, .13] |  | Teacher-cluster bootstrap |
| Specific indirect: exercise self-efficacy | .10 | .022 | [.07, .15] |  | Teacher-cluster bootstrap |
| Specific indirect: peer connectedness | .07 | .020 | [.04, .12] |  | Teacher-cluster bootstrap |
| Total indirect association | .25 | .031 | [.20, .32] |  | Teacher-cluster bootstrap |
| Total association | .49 | .032 | [.43, .55] | < .001 | Cluster-adjusted MLR |

Note. Primary structural paths, direct associations, and model fit are reported from the MLR model with TYPE = COMPLEX and CLUSTER = teacher_id. Specific indirect associations and their confidence intervals are reported from the complementary D2 ML model with TYPE = COMPLEX, CLUSTER = teacher_id, and 5,000 bootstrap draws.

# Supplementary Table S10. Pairwise Contrasts of Specific Indirect Associations

| **Contrast** | **Unstandardized difference** | **Bootstrap SE** | **95% bootstrap CI** | **Decision** |
| --- | --- | --- | --- | --- |
| Exercise self-efficacy minus emotion regulation | .02 | .011 | [-.002, .042] | Not significant |
| Exercise self-efficacy minus peer connectedness | .03 | .012 | [.006, .054] | Significant |
| Emotion regulation minus peer connectedness | .01 | .010 | [-.010, .030] | Not significant |

Note. Estimates, bootstrap SEs, and confidence intervals in this table refer to unstandardized pairwise contrasts defined in D2 MODEL CONSTRAINT. Table 6 reports standardized (STDYX) indirect associations. Requesting STDYX output does not standardize MODEL CONSTRAINT parameters. The ESE-minus-PC contrast excludes zero; the other two contrasts include zero.

# Supplementary Table S11. Construct-Overlap Sensitivity Analysis

| **Parameter** | **Original five-dimension PE predictor** | **Narrow PE predictor (TS + MC + EE)** | **Stability criterion** |
| --- | --- | --- | --- |
| PE -> emotion regulation | .41 | .39 | Direction preserved; magnitude stable |
| PE -> exercise self-efficacy | .39 | .38 | Direction preserved; magnitude stable |
| PE -> peer connectedness | .46 | .44 | Direction preserved; magnitude stable |
| PE -> resilience-based recovery capacity | .24 | .23 | Direction preserved; slightly reduced |
| Indirect via emotion regulation | .08 | .07 | Direction and magnitude stable |
| Indirect via exercise self-efficacy | .10 | .10 | Direction and magnitude stable |
| Indirect via peer connectedness | .07 | .06 | Direction and magnitude stable |

*Note. The narrow predictor excludes Emotional Release and Peer Interaction to address item-content overlap with emotion regulation and peer connectedness.*

# Supplementary Table S12. Sensitivity to Extracurricular Exercise-Frequency Coding

| **Specification** | **Reference/category coding** | **PE -> recovery** | **Stress -> recovery** | **Exercise covariate(s) -> recovery** | **Conclusion** |
| --- | --- | --- | --- | --- | --- |
| Primary model | Ordered categories 1-4; approximately continuous covariate | 0.24 | -0.36 | .11 (SE=.045, p=.014) | Reported benchmark |
| Categorical sensitivity | D1=1 time/week; D2=2-3 times/week; D3=4+ times/week; reference=0 times/week | 0.23 | -0.35 | D1=.09 [.02,.16], p=.012; D2=.12 [.04,.20], p=.003; D3=.10 [.03,.17], p=.006 | Focal paths stable; the pattern is not interpreted as a monotonic dose-response relationship |

Note. The sensitivity analysis evaluates whether the focal association depends on treating the four ordered extracurricular exercise-frequency categories as equally spaced. The 0-times-per-week category served as the reference group. The pattern should not be interpreted as evidence of a monotonic dose-response relationship.

# Supplementary Table S13. Sampling, Data Screening, Missingness, and Clustering Record

| **Record** | **Final report** |
| --- | --- |
| Questionnaires distributed | 900 across four institutions |
| Eligibility exclusions | 28 (3.1%) |
| Response-pattern quality exclusions | 21 (2.3%); union of within-person raw-item SD < .20, cyclical responding across at least 15 consecutive items, or failure of either of the two attention checks |
| Completion-time exclusions | 20 (2.2%); completion time < 480 seconds |
| Final analytic sample | 831 (92.3%); complete-case sample with zero item-level missingness |
| Clustering structure | 58 PE classes nested within 35 teachers; teacher-level clustering accounted for with TYPE = COMPLEX and CLUSTER = teacher_id |
| Collection context | March-May 2025; Xiangyang and Xianning, Hubei Province; convenience and purposive recruitment through intact PE classes and QR-code questionnaires |

Note. The screening categories are mutually exclusive and sequential. No item-level missingness remained in the final analytic sample. The four participating institutions are identified as Institutions A-D in the sample-distribution materials; study locations, author affiliations, ethics information, and funding disclosures are reported elsewhere as appropriate.

# Appendix B. Contextualized Self-Efficacy and Peer Connectedness Items

The following items were contextualized for the university PE setting and are reported in English translation. They were rated on the same 5-point Likert-type scale from 1 = strongly disagree to 5 = strongly agree. The translated wording is provided to make the adaptations transparent and replicable.

| **Code** | **Construct** | **English item** |
| --- | --- | --- |
| ESE1 | Exercise self-efficacy | Even when I have many academic tasks, I am confident that I can complete the required PE activities. |
| ESE2 | Exercise self-efficacy | When physical activities are somewhat difficult, I still believe that I can persist until completion. |
| ESE3 | Exercise self-efficacy | I am confident that I can gradually improve my sport performance through practice. |
| ESE4 | Exercise self-efficacy | Even if I do not perform well at first, I am confident that I can continue participating in physical activity. |
| ESE5 | Exercise self-efficacy | I believe that I can use exercise to help regulate stress or emotions. |
| ESE6 | Exercise self-efficacy | Outside PE class, I am also confident that I can maintain a certain level of exercise habit. |
| PC1 | Peer connectedness | In PE class, I felt that I was part of the class or group. |
| PC2 | Peer connectedness | Interactions with classmates in PE class made me feel accepted. |
| PC3 | Peer connectedness | When PE activities required cooperation, I could feel peer support. |
| PC4 | Peer connectedness | PE class gave me opportunities to know or become closer to more classmates. |
| PC5 | Peer connectedness | Peer interaction in PE class made me feel less isolated. |
| PC6 | Peer connectedness | Participating in physical activities with classmates made it easier for me to relax. |

# Appendix C. Established Scale Versions and Scoring Procedures

Table C1 records the source instrument, the response format used in this study, reverse scoring, and score computation. The harmonized 1-5 agreement response format differs from the original metric of some source instruments and is therefore reported as a study-specific adaptation.

Full item wording for the established instruments is not reproduced here. Table C1 identifies the Chinese validation sources, administered response anchors, reverse scoring, and score construction.

The PE Course Experience items listed in Appendix A/Supplementary Table S1 and the Exercise Self-Efficacy and Peer Connectedness items listed in Appendix B are the contextualized items used for this study and should be further evaluated through reliability, CFA, and measurement invariance testing in future samples.

# Supplementary Table C1. Established Scale Versions, Adaptations, and Scoring

| **Construct** | **Source/version** | **Items** | **Administered response format** | **Reverse scoring** | **Score computation and reporting boundary** |
| --- | --- | --- | --- | --- | --- |
| Resilience-based recovery capacity | CD-RISC-10; Wang et al. (2010); Campbell-Sills and Stein (2007) | 10 | 1 = strongly disagree to 5 = strongly agree | None | Mean of 10 items; adaptation from the source response metric. Interpreted as resilience-based adaptive capacity, not immediate recovery state. |
| Perceived stress | PSS-10; Lu et al. (2017); Cohen and Williamson (1988) for the original construct | 10 | 1 = strongly disagree to 5 = strongly agree | Items 4, 5, 7, and 8; recoded as 6 - response | Mean of 10 items after reverse scoring; harmonized agreement anchors are a study-specific adaptation. |
| Emotion regulation | ERQ cognitive reappraisal; Wang et al. (2007); Gross and John (2003) | 6 | 1 = strongly disagree to 5 = strongly agree | None within the reappraisal subscale | Mean of 6 items; the original ERQ response metric was harmonized to five points. |

Note. The established instruments were administered in Chinese and adapted to a common 1-5 agreement format. The validity of the source versions does not automatically transfer to this changed response format.

# Appendix D. Mplus Analysis Specifications and Reproducibility Notes

Appendix D describes the primary Mplus analysis specifications used in this revision. D1 describes the primary MLR structural model with TYPE = COMPLEX and CLUSTER = teacher_id; D2 describes the complementary ML bootstrap model with 5,000 draws; and D3 describes the 15-item PE common-latent-factor sensitivity model. D1-D3 are analytic specifications provided to improve transparency and reproducibility and should not be interpreted as a complete executable archive of every supplementary analysis.

D1 and D2 use item-level indicators and the observed extracurricular-exercise-frequency covariate. D3 uses the 15 PE indicators and an orthogonal method factor with freely estimated item loadings and fixed variance. PSS items 4, 5, 7, and 8 must be reverse-scored in the analysis data before D1 or D2 is run. The model labels in D2 define unstandardized indirect associations and pairwise contrasts; requesting STDYX output does not standardize MODEL CONSTRAINT parameters.

Specific indirect associations are reported on the STDYX scale, while pairwise contrasts are reported on the unstandardized indirect-association scale. The study does not interpret these cross-sectional associations as evidence of temporal or causal ordering.

# **Supplementary References**

Campbell-Sills, L., and Stein, M. B. (2007). Psychometric analysis and refinement of the Connor-Davidson Resilience Scale (CD-RISC): validation of a 10-item measure of resilience. J. Trauma. Stress 20, 1019-1028. doi: 10.1002/jts.20271

Cohen, S., and Williamson, G. (1988). "Perceived stress in a probability sample of the United States," in The Social Psychology of Health: Claremont Symposium on Applied Social Psychology, eds. S. Spacapan and S. Oskamp (Newbury Park, CA: Sage), 31-67.

Gross, J. J., and John, O. P. (2003). Individual differences in two emotion regulation processes: implications for affect, relationships, and well-being. J. Pers. Soc. Psychol. 85, 348-362. doi: 10.1037/0022-3514.85.2.348

Lu, W., Bian, Q., Wang, W., Wu, X., Wang, Z., and Zhao, M. (2017). Chinese version of the Perceived Stress Scale-10: A psychometric study in Chinese university students. PLoS ONE 12:e0189543. doi: 10.1371/journal.pone.0189543

Polit, D. F., Beck, C. T., and Owen, S. V. (2007). Is the CVI an acceptable indicator of content validity? Appraisal and recommendations. Res. Nurs. Health 30, 459-467. doi: 10.1002/nur.20199

Wang, L., Liu, H., Li, Z., and Du, W. (2007). Reliability and validity of emotion regulation questionnaire Chinese revised version. China J. Health Psychol. 15, 503-505.

Wang, L., Shi, Z., Zhang, Y., and Zhang, Z. (2010). Psychometric properties of the 10-item Connor-Davidson Resilience Scale in Chinese earthquake victims. Psychiatry Clin. Neurosci. 64, 499-504. doi: 10.1111/j.1440-1819.2010.02130.x
